# Resistance of bonded premolars to four artificial ageing models post enamel conditioning with a novel calcium-phosphate paste

**DOI:** 10.4317/jced.56764

**Published:** 2020-04-01

**Authors:** Ali I. Ibrahim, Noor R. Al-Hasani, Van P. Thompson, Sanjukta Deb

**Affiliations:** 1 Centre for Oral, Clinical and Translational Sciences, Faculty of Dentistry, Oral & Craniofacial Sciences, King’s College London, London, UK; 2 Department of Orthodontics, College of Dentistry, University of Baghdad, Baghdad, Iraq; 3 Institute of Pharmaceutical Science, King’s College London, London, UK; 4 Department of Basic Sciences, College of Dentistry, University of Baghdad, Baghdad, Iraq

## Abstract

**Background:**

This *in vitro* study compares a novel calcium-phosphate etchant paste to conventional 37% phosphoric acid gel for bonding metal and ceramic brackets by evaluating the shear bond strength, remnant adhesive and enamel damage following water storage, acid challenge and fatigue loading.

**Material and Methods:**

Metal and ceramic brackets were bonded to 240 extracted human premolars using two enamel conditioning protocols: conventional 37% phosphoric acid (PA) gel (control), and an acidic calcium-phosphate (CaP) paste. The CaP paste was prepared from β-tricalcium phosphate and monocalcium phosphate monohydrate powders mixed with 37% phosphoric acid solution, and the resulting phase was confirmed using FTIR. The bonded premolars were exposed to four artificial ageing models to examine the shear bond strength (SBS), adhesive remnant index (ARI score), with stereomicroscopic evaluation of enamel damage.

**Results:**

Metal and ceramic control subgroups yielded significantly higher (*p* < 0.05) SBS (17.1-31.8 MPa) than the CaP subgroups (11.4-23.8 MPa) post all artificial ageing protocols, coupled with higher ARI scores and evidence of enamel damage. In contrast, the CaP subgroups survived all artificial ageing tests by maintaining adequate SBS for clinical performance, with the advantages of leaving unblemished enamel surface and bracket failures at the enamel-adhesive interface.

**Conclusions:**

Enamel conditioning with acidic CaP pastes attained adequate bond strengths with no or minimal adhesive residue and enamel damage, suggesting a suitable alternative to the conventional PA gel for orthodontic bonding.

** Key words:**Enamel etching, calcium phosphate, bracket bond strength, adhesive residue, enamel damage.

## Introduction

Conventional orthodontic bracket bonding techniques involve enamel pre-treatment with 37% PA gel to ensure secure attachment of the bracket to the enamel surface. Etching with 37% PA removes the outermost enamel layer, creates surface irregularities and micro-pores to enhance adhesive material infiltration resulting in optimal micro-mechanical retention with the enamel surface. Although this approach ensures high bracket bond strengths, it frequently results in enamel cracks and fractures due to the higher debonding force required to remove the brackets after treatment (1). This problem is exacerbated when using the aesthetic ceramic brackets because of their rigidity and lack of deformation during removal, with greater amount of remnant adhesive left on enamel and the subsequent prolonged chair-side time required to clean and polish the tooth surface (2). Attempts to use different concentrations of other acids e.g. pyruvic, maleic, and citric acids have been found to be less effective than phosphoric acid, thus have not been employed in orthodontic bonding (3). On the other hand, alternative enamel conditioning methods such as laser etching and the use of self-etch primers resulted in bracket bond strengths suitable for clinical performance; however, could not eliminate enamel damage and the amount of remnant adhesive left on tooth surface (2,4).

Recently, a novel etchant system for orthodontic bracket bonding (International Patent: WO 2018/011338 A1) was developed to reduce the bracket bonding iatrogenic effects such as enamel demineralization and/or enamel damage encountered at bracket debonding step following the completion of treatment. Etchant pastes made of calcium-phosphate (CaP) powders mixed with 37% phosphoric acid were developed for the first time to simultaneously etch and re-mineralize the enamel surface before bracket bonding (5). This approach facilitated the preferable bracket debonding failure at the enamel-adhesive interface, with no or minimal adhesive remnants after debonding, thus saving chair-side time and cost at the post debonding clean-up stage. The established enamel-adhesive-bracket interfaces following this technique survived *in vitro* 24 h water storage and post 5000 thermo-cycles successfully, yielding clinically accepTable SBS values. However, the role of other complex intraoral conditions represented by the continuous changes in pH, humidity, occlusal forces and eating habits should be investigated before attempting the novel CaP etchant paste in a clinical trial.

Therefore, the aim of this study is to expose metal and ceramic brackets bonded to CaP paste-treated enamel surfaces to further reproducible, aggressive artificial ageing models to help predict the behaviour and lifespan of the novel bonding system during clinical ageing, in comparison with the standard etch-and-rinse approach using 37% PA gel. The artificial ageing models involve separate exposure to prolonged acidic challenge, water storage, and 5000 fatigue cyclic loading.

## Material and Methods

Metal and ceramic brackets were equally bonded to 240 extracted premolar teeth collected from orthodontic patients (15-30 years) after acquiring ethical approval from the National Research Ethics Service Committee London-Riverside (REC Reference 14/LO/0123). After extraction, the teeth were cleaned in running water, then stored in a 1% chloramine-T trihydrate bacteriostatic/bactericidal solution for one week followed by storage in distilled water (ISO/TS 11405:2015). Teeth were examined under stereomicroscope (x10 magnification) for selecting intact buccal enamel surface without cracks and caries, with no history of previous orthodontic or bleaching treatments (2).

Equimolar amounts of beta-tricalcium phosphate (β-TCP) and monocalcium phosphate monohydrate (MCPM) powders (Sigma-Aldrich, UK) were mixed with 37% phosphoric acid solution (Sigma-Aldrich, UK) in a powder-to-liquid ratio of 0.8:1 for preparing acidic CaP etchant pastes (5). The assigned powder and liquid were mixed using a stainless steel spatula on a glass slab for 30 seconds until a homogenous workable paste was obtained. The pH of the resulting paste was immediately measured by a flat-end electrode using a digital pH meter (Oakton, Singapore). Fourier transform infrared (FTIR) spectroscopy (ATR accessory, spectrum one, Perkin Elmer, USA) was used to examine the resultant phase of the prepared CaP paste at 2 time points through identifying the distinctive functional groups. A small amount of the prepared paste was examined with FTIR within one minute from the start of mixing, whilst the rest of the paste was kept in a glass vial for 24 h and ground with a mortar and pestle after complete setting to get CaP powder for the second FTIR examination. FTIR-ATR diamond crystal was used, with a resolution of 4 cm-1, eight background scans and a spectral range from 4000 cm-1 to 650 cm-1.

-Preparation and Classification of Samples 

**Figure 1:**
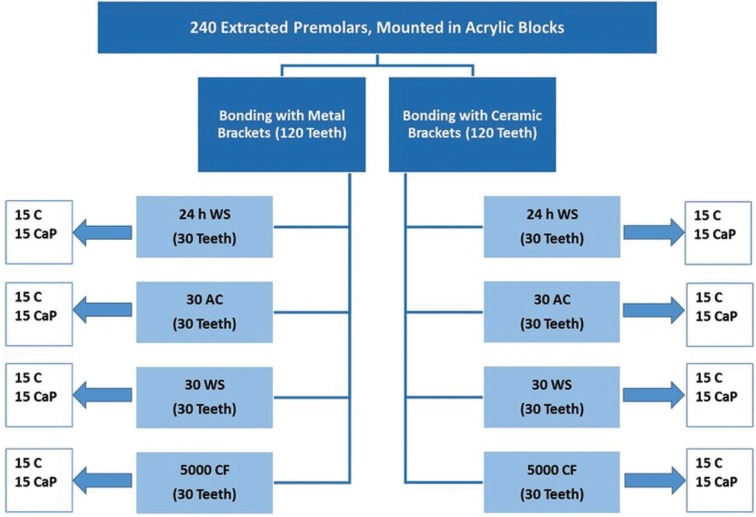
A schematic illustration of the study design. Bonded teeth with either metal or ceramic brackets were exposed to 4 separate artificial ageing tests: 24 hours water storage (24 h WS), 30 days acid challenge (30 AC), 30 days water storage (30 WS), and 5000 cycles of cyclic fatigue (CF). Each main metal or ceramic group was subdivided into two subgroups according to the type of enamel etchant: control (C) using 37% PA gel and experimental etchant paste (CaP).

Two hundred and forty extracted human premolars were randomly allocated into two main groups to be bonded with 120 metal (stainless steel) and 120 ceramic (sapphire) brackets. Each of the main groups was further subdivided into two subgroups according to the type of enamel etchant and subjected to four separate artificial ageing tests as demonstrated in Figure 1.

Teeth were mounted vertically inside acrylic blocks using rubber moulds (14 mm length x14 mm width x17 mm depth) and aligned using the analyzing rod of a surveyor to ensure a debonding force running parallel to the bonded bracket base. Then self-cure clear acrylic (Oracryl, Bracon, UK) was poured around the tooth up to about 1 mm apical to the level of cemento-enamel junction. The mounted teeth were subsequently stored in distilled water (DW) at lab temperature until bonding.

-Enamel conditioning and bracket bonding procedures 

Two types of pre-adjusted upper premolar brackets were used: metal (Pinnacle, stainless steel, MBT, slot 0.022x0.028 inch, Orthotechnology, USA) and ceramic (NeoCrystal, monocrystalline sapphire, MBT, slot 0.022x0.028 inch, Henry Schein, USA). Transbond XT light-cure primer and adhesive (3M Unitek, Monrovia, California, USA) were used for all brackets bonding. The conventional etch-and-rinse protocol (6) was followed to bond all brackets starting with enamel surface polishing (10 s) using pumice slurry and rotary rubber cups, water irrigation (10 s) and oil-free air dryness (10 s). Etching of the buccal enamel surface was achieved for 30 seconds using either 37% PA etchant gel (Resilience, Ortho Technology, USA) for the control subgroups, or CaP etchant paste for the experimental subgroups, followed by water irrigation (20 s) and dryness (20 s). A thin layer of Transbond XT light-cure primer was applied onto the etched surface and spread by air-jet (3 s), then loading the bracket base with Transbond XT light-cure composite adhesive to be attached to the enamel surface. LED Light-curing (3M ESPE, Elipar DeepCure-S, USA, 1470 mW/cm2 light intensity) was applied for 20 s (10 s on each mesial and distal side) according to the manufacturer instructions. The bonded teeth were exposed to 4 separate artificial ageing models.

-Artificial ageing models

1- Standard 24 h Water Storage (24 h WS): bonded brackets stored in DW for 24 h at 37°C before bracket debonding.

2- Thirty-days Acid Challenge (30 AC): bonded brackets stored in DW for 30 days at 37°C with daily 15 minutes intermittent acidic attack. Samples were first immersed in DW at 37°C for 24 h before conducting the AC experiment. An acidic solution (pH=2.5) of 250 ml was prepared by adding 7.5 ml of 1M HCl with DW. All samples were subjected to acidic attacks by immersing in the acidic solution using a protocol (7) of 3 sessions per day, 5 min each, with equal intervening intervals (2 hours) for 30 days. Samples were kept in DW (pH=6) at 37°C for the rest of the day to simulate the wet oral environment. DW and acidic solutions were replenished after each session, and samples were washed and air dried before and after each session.

3- Thirty-days Water Storage (30 WS): bonded brackets stored in DW for 30 days at 37°C in order to rule out any potential effect of prolonged water storage on bond strength accompanying the 30-days acidic attack; with daily refreshment of the DW.

4- Cyclic Fatigue (5000 CF): bonded brackets subjected to 5000 cyclic loadings after 24 h water storage at 37°C. To run the CF test, the acrylic block part of each specimen was fixed within the grips of fatigue machine (Bose, EnduraTec, USA) using a metal clamp. A hardened stainless steel edge (0.5 mm) was adjusted to apply cyclic occluso-gingival forces to the base of the bracket. Compressive fatigue limits were determined according to the “staircase” method, which provides a good measure of the mean fatigue limit and permits calculation of the standard deviation of that mean (8). According to this method, fatigue testing starts with an initial load value that represents a fraction of the corresponding mean static shear bond strength (SSBS). The tests were conducted sequentially, with the maximum applied stress in each succeeding sample being increased or decreased by a fixed amount, usually 5% of the initial load applied, according to whether the previous stress resulted in a failure or non-failure of the bracket. The procedure of increasing the maximum stress by this increment following a test in which no failure occurred, and decreasing the stress by the same increment following a failure, continued for each succeeding specimen until all the specimens of a specific subgroup were evaluated. Fatigue testing of each subgroup was started with an initial load value that represents 60% of the mean SSBS of the corresponding subgroup obtained by debonding carried out after 24 h immersion of the bonded teeth in DW at 37°C. The cyclic testing was achieved at a frequency of 1 Hz, which corresponds to the reported oral chewing frequency (9). The analysis of the data was based on the least frequent event (bracket failure or non-failure). To determine the mean fatigue limit and its standard deviation, the data were arranged in a descending manner, starting from the highest to the lowest strength. The lowest stress level at which a failure or non-failure occurs is denoted by i = 0, the next i = 1, and so on. The number of events (failure or non-failure) at the given stress level equals ni. The mean fatigue limit, X, and its standard deviation, SD, were calculated according to equations [1] and [2] respectively.

X= XO + [d (A/N ± 1/2)] ……[1]

SD= 1.62 d ((NB-A²)/N² + 0.029) ……[2] 

The XO is the lowest stress level at which the least frequent event occurred in the analysis, and d is the stress increment (taken from the initial fatigue load applied) employed in the sequential tests. The other constants are defined by: N = ∑ ni , A = ∑ ini , and B = ∑ i2ni. In formula [1], the positive sign was used when the analysis is based on bracket non-failure, and the negative sign was used when failures were considered (8).

Bracket debonding for SBS and adhesive remnant index (ARI) assessment

Except for the cyclic fatigue experiment where cyclic shear forces were applied as aforementioned, a static shear force test was applied following each artificial ageing experiment to examine the SBS, ARI and enamel damage upon bracket removal. A chisel on a universal testing machine (Instron, Model 5569A, USA) applied an occluso-gingival load vertically at the bracket base with a crosshead speed of 0.5 mm/min. The SBS values were calculated in MPa by dividing the load at failure by the bracket base surface area. The debonded enamel surfaces were examined with a stereomicroscope (MEIJI, EMZ-TR, Japan) under x10 magnification for enamel damage, including enamel fracture (EF) or cracking (EC), and the amount of remnant adhesive left which was scored according to the ARI scoring system (10).

-Statistical Analysis

Using G-power version 3.1.9.2, a sample of 15 specimens per subgroup was required for an experiment to detect a significant difference in SBS between subgroups with an effect size of 0.35 and 80% power 2-tailed test at 5% level of significance. SPSS statistical software (version 26, SPSS Inc., IBM, USA) was used at a level of significance *p*> 0.05. Data were tested for normality using Histogram/Q-Q plots/Shapiro-Wilk tests. Independent samples t-test was conducted for parametric data analysis (SBS), whilst Mann-Whitney test was used for non-parametric data (ARI scores).

## Results

-Characterization of the CaP etchant paste

**Figure 2:**
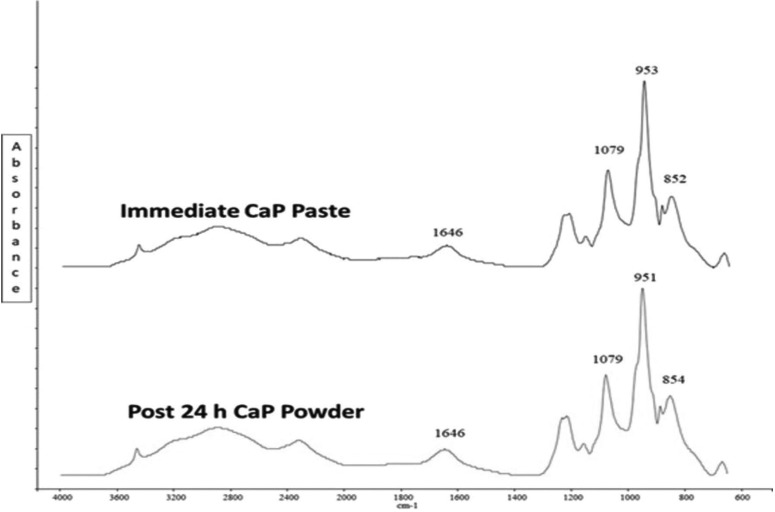
FTIR spectra of the CaP formulation at two time points, depicting almost identical 4 distinctive bands. Both the immediately mixed paste and post-24 h ground powder elicited a P-O(H) peak at around 853 cm-1, two P-O peaks at around 952 cm-1 and 1079 cm-1, and the H-O-H bending mode of water at 1646 cm-1 indicating monocalcium phosphate monohydrate formation.

The FTIR results of the CaP etchant pastes showed identical spectra of the immediate paste examination and powder examination after 24 h storage. The spectra identified peaks position and intensity that were close to the distinctive bands of MCPM, associated with the stretching mode of the P-O(H) group, the two P-O stretching peaks, and the H-O-H bending mode of water (Fig. 2).

Assessment of bond strength and adhesive remnant index post artificial ageing tests

The static SBS and ARI scores frequency distribution of the control 37% PA gel and CaP etchant paste subgroups obtained on metal and ceramic bracket debonding following 24 h WS compared with 30-days AC, and in comparison with 30-days WS are shown in  Table 1 and  Table 2 respectively. The daily exposure of the bonded premolars to 3 sessions of acidic attacks over 30-days period resulted in a drop in the SBS of the control and CaP subgroups following both metal and ceramic bracket debonding. However, the reduction was statistically non-significant in SBS, with a non-significant change in the distribution of ARI scores (Table 1). In contrast, the 30-days WS model yielded closer SBS and ARI scores to those of the 24 h WS with statistically non-significant alterations for both metal and ceramic subgroups (Table 2).

**Table 1:**
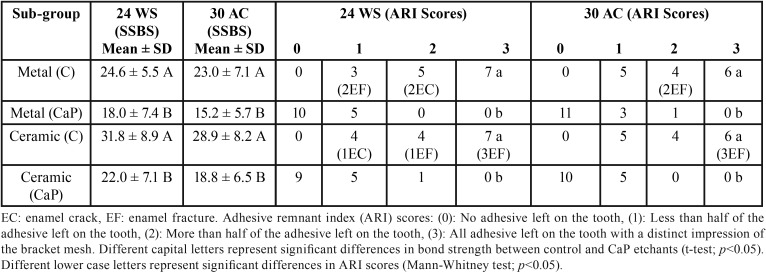
Metal and ceramic bracket debonding after 24 h water storage (24 WS) and 30-days acid challenge (30 AC). The static shear bond strength (SSBS) and remnant adhesive of the control and experimental (CaP) etchants were compared at the two bracket debonding time points.

**Table 2:**
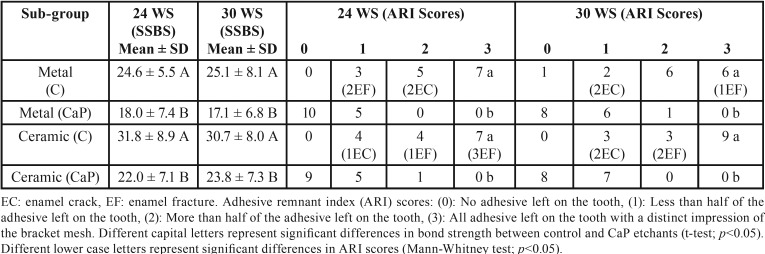
Metal and ceramic bracket debonding after 24 h water storage (24 WS) and 30-days water storage (30 WS). The static shear bond strength (SSBS) and remnant adhesive of the control and experimental (CaP) etchants were compared at the two bracket debonding time points.

Following the artificial ageing tests, the metal and ceramic control (C) subgroups exhibited significantly higher SBS mean values than the experimental CaP subgroups. In addition, enamel etching with the control PA gel resulted in significantly higher ARI scores (majority of scores 2 and 3) than etching with the CaP paste (mainly scores 0 and 1); i.e. the CaP subgroups showed less remnant adhesive on enamel following both metal and ceramic brackets debonding. After stereomicroscopic examinations, 9 out of 45 metal control samples elicited enamel damage [4 enamel cracks (EC) and 5 enamel fractures (EF)], whilst 12 out of 45 ceramic control samples showed enamel damage [3 EC and 9 EF] at bracket debonding. In contrast, no enamel damage was observed at both metal and ceramic brackets debonding of all CaP paste subgroups.

An illustration of the staircase method of analyzing fatigue testing outcomes to calculate the mean fatigue limit and the standard deviation is shown in Table 3, using results of the metal control subgroup as an example. Table 4 shows the cyclic shear bond strength (CSBS) results of fatigued metal and ceramic subgroups obtained following 5000 cycles. The Table also demonstrates percentages of fatigue-induced drop in bond strength (fatigue ratio), independent samples t-test comparisons between the static SBS obtained from the standard 24 h WS experiment and the CSBS. Fatigue testing resulted in significant reductions in bond strength of all metal and ceramic subgroups.

**Table 3:**
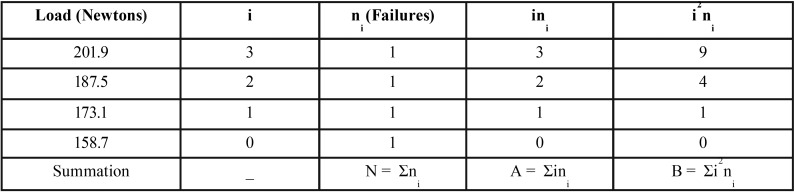
Analysis of fatigue data using the staircase method. The loads obtained from fatigued metal brackets of the control subgroup after 5000 cycles are shown as an example, arranged in a descending manner. The analysis is based on the least frequent event (4 bracket failures): the lowest stress level at which a failure or non-failure occurs is denoted by i = 0, the next i = 1, and so on. The number of events (failure or non-failure) at the given stress level equals ni. The N, A and B are required to calculate the mean fatigue limit and standard deviation.

**Table 4:**
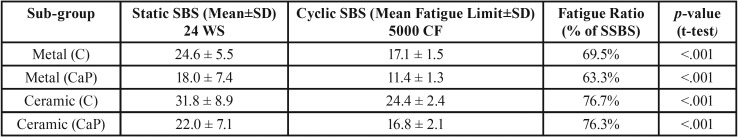
Shear bond strength (SBS) of metal and ceramic bracket subgroups post 5000 cyclic fatigue loadings (5000 CF). The fatigue ratio represented the equivalent percentages of cyclic SBS to the static SBS obtained from the standard 24 h water storage experiment. The t-test revealed significant reductions in bond strength of all subgroups after 5000 CF.

**Figure 3:**
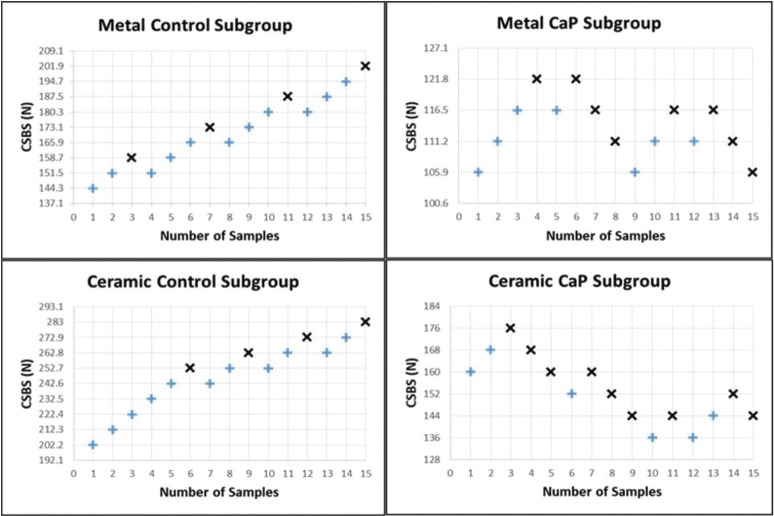
Fatigue staircase cyclic shear bond strength (CSBS) of premolars etched with 37% PA gel (control) or CaP paste, and bonded with metal or ceramic brackets. The fatigue analysis for each subgroup is based on the least frequent event [bracket failure (x) or non-failure (+)] throughout the test.

Enamel etching with 37% PA gel (control metal and ceramic subgroups) resulted in a greater resistance to cyclic fatigue loading compared to CaP paste subgroups. This was represented by the higher mean fatigue limits and greater percentages of static SBS (fatigue ratio) attained by the control subgroups than those of CaP subgroups. The ARI scores could not be assessed following cyclic fatigue testing because of the high frequency of bracket non-failures, which impede the proper examination of adhesive material. The plots of staircase cyclic fatigue outcomes of all metal and ceramic subgroups are presented in Figure 3, which depicts the frequency of events required for data analysis. Determination of the mean fatigue limit and its standard deviation for each subgroup was premised on the least frequent event (failure or non-failure of brackets).

## Discussion

Orthodontic therapy contributes significantly to improving oral health and facial aesthetics; however, it is frequently associated with a wide range of enamel damage. Enamel mineral loss occurs initially when pumicing and etching the enamel surface to induce demineralization and generate a micro porous surface to provide micro-mechanical retention between the enamel and adhesive material. Further enamel demineralization and white spot formation can occur throughout the prolonged treatment duration (an average of 12-18 months) (11), whilst enamel fracture or cracking is frequently encountered at the bracket removal step following completion of treatment (1). Moreover, additional trauma to the enamel is still expected after appliance removal due to the adhesive remnants left on enamel, which require extra chair-side time to be removed by traumatic rotary burs that have been shown to result in ineviTable enamel scratching (12). Accordingly, a recent *in vitro* study focused on developing a novel re-mineralizing CaP etchant paste that yielded satisfactory bracket bond strengths, together with minimal adhesive residue and damage to tooth structure (5). However, an ideal orthodontic bonding system must reproduce good bond strength, support masticatory loads, resist intraoral fluctuations in moisture, pH and temperature, and be easily removable at the end of the treatment without causing injury to the tooth surface. Therefore, the current study is complementary to the previous investigation, aiming at exposing CaP paste-etched premolars to further aggressive laboratory testing to establish a robust platform for conducting a clinical trial.

The prevalent practice in orthodontics is to etch the enamel with 35-40% phosphoric acid before bracket bonding. The bond strengths obtained via this protocol are usually high, ranging between 10-35 MPa with usually higher values being attained by ceramic than metal brackets (13), but frequently associated with significant remnant adhesive left on enamel, chipping or cracking of enamel at bracket debonding (6,14). The bond strengths of all metal and ceramic subgroups in this study are within the confines of this range. The higher bond strengths elicited by ceramic brackets can be attributed to the facts that the ceramic bracket translucency allows for enhanced polymerization of the adhesive through a better transmission of curing light, and that coating of sapphire bracket base with zirconia and brushite-ceramic powders creates millions of undercuts for extra mechanical locking with the adhesive. Moreover, the high elastic modulus of a ceramic bracket does not allow it to deform the bonded interface as occurs with the metal bracket during loading (6,15,16).

The control metal and ceramic subgroups exhibited significantly higher SBS and ARI scores than the subgroups etched with the CaP paste prepared from comparably strong phosphoric acid solution. Mixing CaP powders with 37% PA solution (pH 0.8) raised the pH in the resulting paste to 1.4, which was monocalcium phosphate monohydrate (MCPM) as confirmed by FTIR; similar to the acidic paste developed in the previous work (5). Acid-etching of enamel creates surface micro-pores by dissolving calcium and phosphate ions, which re-precipitate as MCPM following enamel conditioning with PA of concentrations exceeding 27% (17). The volume and low solubility of calcium phosphate crystallites encourage their stagnation and resistance to water irrigation, which can adversely affect the bond strength (18). Since the etchant paste is already made of MCPM, more crystallites precipitation is expected to take place, hence impeding the complete resin infiltration into the micro-pores and yielding lower bond strength (5). However, the mean bond strengths obtained from CaP paste-etched subgroups ranged between 11.4-23.8 MPa, which were above the suggested range of 6-10 MPa as a lower limit for acceptable clinical performance (1,5). Moreover, this was accompanied with the advantage of significantly lower ARI scores, which means leaving minimal adhesive residue with almost unblemished enamel surfaces as compared with the control subgroups.

The most commonly used artificial ageing model to examine the degradation of tooth-adhesive interface is by ageing in water, which is thought to result in hydrolysis and infiltration into the adhesive material to weaken the mechanical properties of its polymer matrix (19,20). The bond strength of clinically bracketed teeth starts to experience the wet oral environment shortly after the bracket bond-up procedure. It has been found that polymerization of resin composites is adversely affected by moisture, and the detrimental effect of water uptake on bond durability is most evident during the first 24 h of inception of polymerization, after which changes in bond strength are non-significant (21). Therefore, water storage of bonded samples for 24 h at 37°C has been a standard protocol before launching further artificial ageing tests (22). In this study, the 30-days water storage results were very close to those of 24 h WS experiment as demonstrated by all metal and ceramic subgroups. This finding agrees with previous studies which reported that robust enamel-adhesive interfaces established following the conventional etch-and-rinse or self-etch approach could survive various water storage durations (30 days to 2 years) without significant changes in SBS and ARI scores in comparison with 24 h WS (22,23). On the other hand, the bond strengths of both metal and ceramic subgroups determined after 30-days acid challenge were lower than the 24 h WS outcomes. It has been reported that consuming acidic drinks during orthodontic treatment can weaken bracket retention and contribute to bond failure by softening the enamel and/or degradation of the adhesive resin material with evidence of leaching of its filler content (24,25). The complexity of the enamel surface is influenced by both acid concentration and duration of acid exposure and to mimic a clinical scenario, an aggressive acid challenge protocol was adopted in this study that assumes an orthodontic patient consuming a highly acidic drink (pH 2.5) 3-times/day with a consumption period of 5 minutes (7,25) for a duration of 30 days which is recognized enough to investigate the enamel demineralization/re-mineralization potential *in vitro* (26). The findings of this study concur with previous studies that used various commercial acidic drinks (e.g. Coca Cola and Sprite, pH range of 2.4-4.3) and showed that acidic attack reduced the SBS, but was above the clinically acceptable range of 6-10 MPa (7,24,25).

Regarding the non-significant changes in the ARI scores distribution demonstrated by all subgroups when comparing the results of acid challenge, 30-days WS experiments to those of 24 h WS, the control subgroups findings agree with the literature as enamel etching with 37% PA and bonding with a highly filled composite resin (e.g. Transbond XT) has been found to yield enamel-adhesive-bracket joints that could maintain a consistent failure behavior following different ageing protocols (3,13). In contrast, enamel etching with the CaP paste introduced a weaker enamel-adhesive than bracket-adhesive interface resulting in a consistent predominance of scores 0 and 1 bracket failure mode throughout all artificial ageing tests. It has been explained before (5) that the CaP paste rationale was based on inducing chemical changes at the enamel-adhesive interface through a milder etching effect with enhancement of CaP re-precipitation.

Brackets in the oral environment are subjected to cyclic loads caused by mastication, occlusion, orthodontic wires and elastics. Although these cyclic stresses could be of a lower magnitude than the static bond strength of a bonded bracket, repetitive subcritical forces occurring throughout the treatment period can cause adhesive micro-fractures and eventual breakdown of the bracket through a condition referred to as fatigue (8). Compared to static SBS, the cyclic SBS provides more realistic information about a material’s long-term performance in the clinical situation (27). The staircase method used to analyze fatigue data provides a feasible and reproducible experimental protocol for the standardization of fatigue studies, by incorporating fatigue loads of magnitudes capable of crack initiation. Fatigue cracks initiation seems to depend on inhomogeneous stress distribution within the bracket-adhesive-substrate complex and appears more likely at magnitudes of cyclic loading higher than 40% of the static SBS, followed by subsequent crack growth and inhomogeneous fracture resistance (9). In the present study, fatigue testing of the enamel-adhesive-bracket complex affected the bond strength outcomes of all subgroups adversely in comparison with the results of 24 h WS experiment. This finding can be attributed to the use of a relatively high initial cyclic loading (60% of the SSBS) and cycle number (5000 cycles), being the highest values ever used in bracketed teeth fatigue studies (9,27), in which a significant drop in SBS was reported after fatigue testing. The control subgroups showed less CSBS reductions in comparison to the CaP subgroups, as indicated by maintaining higher percentages of SSBS (Control= 69.5-76.7% versus CaP= 63.3-76.3%). The known strong etching effect of conventional 37% PA gel, compared to a milder etch pattern induced by the CaP paste accounts for this outcome. The use of percentages reflects the fatigue ratio, i.e. ratio of the mean CSBS to the mean SSBS, which provides helpful information as a predictor of the survival behavior of materials and facilitates comparison with previous reported studies (9,27). The CaP subgroups yielded fatigue ratios between 0.63-0.76, which are in line with the findings of fatigue studies on clinically successful dental adhesive materials reporting fatigue ratios of 0.52-0.70 following etching with conventional 37% PA (8,28,29) indicating the potential effectiveness of using CaP pastes clinically.

Regarding enamel damage (enamel crack and enamel fracture) upon bracket debonding, etching with 37% PA gel resulted in 23% enamel damage, which could not be encountered with CaP paste-etched samples. Studies have reported enamel damage of 15-40% of teeth on bracket debonding following the etch-and-rinse approach using 35-40% PA gel (1,6,12). It has been shown that the risk of enamel damage upon debonding increases as the bracket bond strength exceeds 14 MPa (mean tensile strength of enamel) since excessive bond strengths entail extra force application to debond a bracket, increasing the chance of damage to the enamel (1,2,5). Introducing a relatively weak enamel-adhesive joint following etching with a CaP paste accounts for maintaining a safe bracket debonding in spite of the attainment of SBS above 14 MPa in this study, perhaps as a result of limiting the depth of penetration of the resin into the enamel surface (5). This facilitates the preferable debonding failure at enamel-adhesive interface with the advantage of saving chair-side time and cost at the post debonding clean-up stage. Therefore, an adequate bond strength that allows function over the period of orthodontic treatment is an option when the procedure can lead to unblemished enamel surface with no or minimal adhesive remains and definitely outweighs the unnecessarily traumatic high bond strength.

## Conclusions

Within the limits of this study, the complementary *in vitro* testing using long-term ageing models yielded metal and ceramic bracket bond strengths above the suggested minimum requirements for clinical performance. Accordingly, the intra-oral effectiveness of the novel CaP etchant paste needs to be assessed by conducting a clinical trial in the next step.
